# Supplementary material for: Mechanism of trinucleotide repeat expansion by MutSβ-MutLγ and contraction by FAN1
Source: Nat Commun. 2025 Oct 27;16:9445. doi: 10.1038/s41467-025-64485-w (PMC12559329; doi:10.1038/s41467-025-64485-w)
Supplement: Supplementary file 1 — Supplementary Information [file 41467_2025_64485_MOESM1_ESM.pdf]

Supplementary information for:

Mechanism of trinucleotide repeat expansion by MutSβ-MutLγ and contraction by FAN1

Issam Senoussi, Valentina Mengoli, Arianna Cerana, Andrea Rinaldi, Andrés Marco, Giordano Reginato, Simone G Moro, Ananya Acharya, Megha Roy, Akshay Jayachandran, Elda Cannavo, Ilaria Ceppi, Petr Cejka

Table of contents:

Supplementary Figures 1-8 with legends
Supplementary Tables 1-7 with legends

Supplementary Figure 1

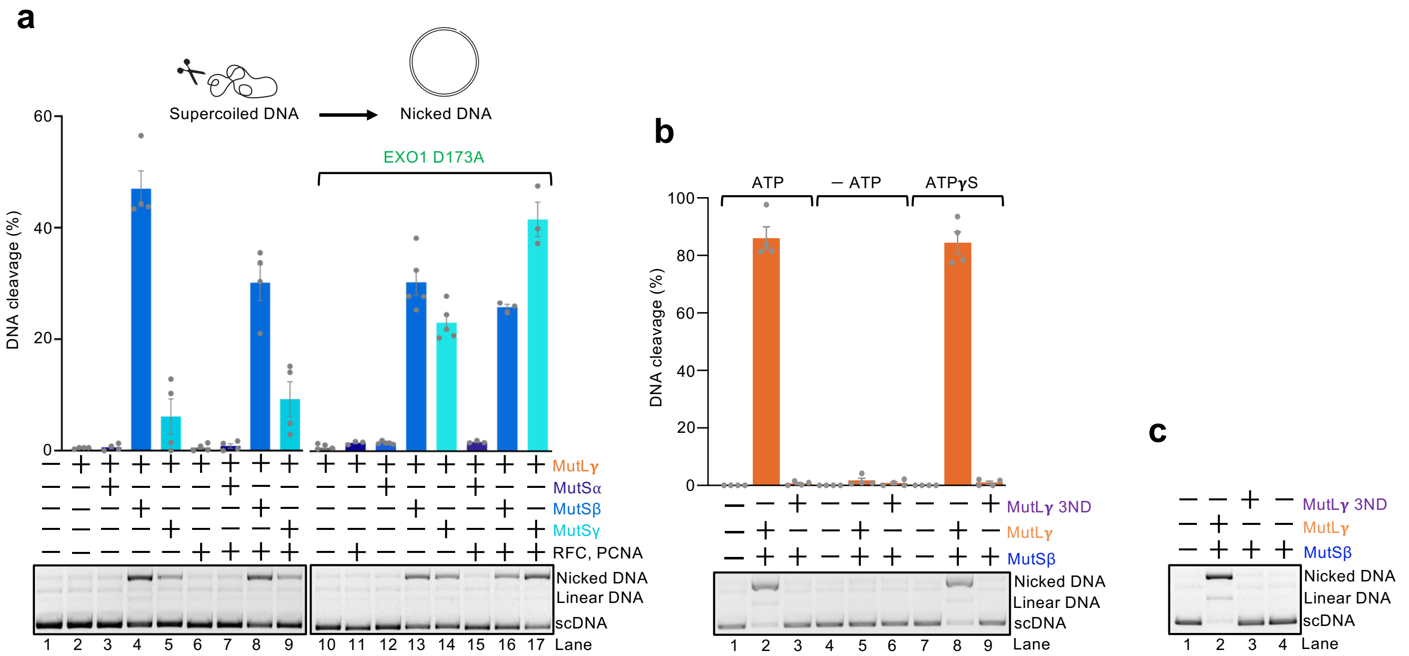

Supplementary Figure 1. Nuclease assays with indicated MutS- and MutL-heterodimer proteins and negatively supercoiled DNA. a, Nicking assays with MutLγ and the indicated co-factors on 5.6-kb-long supercoiled DNA (scDNA). Top, averages shown; error bars, s.e.m.; n=4 independent experiments, except for lanes 12, 13, 14 (n=5) and lanes 11, 15, 16, 17 (n=3). Bottom, representative experiments. The panel shows the same data as in Fig. 1d with additional controls. Values are plotted again as reference. Nuclease-deficient EXO1 D173A contains the D173A point mutation. The reaction products were separated by electrophoresis in the presence of GelRed. **b**, Nicking assays with 5.6-kb-long scDNA, the indicated proteins, and with ATP, without ATP or with ATPγS. Nuclease-deficient MutLγ 3ND contains D1223N, Q1224K and E1229K point mutations in MLH3 endonuclease domain. Top, averages shown; error bars, s.e.m.; n=4 independent experiments. Bottom, representative experiment**.** The reaction products were separated by electrophoresis in the presence of GelRed. **c**, Nicking assays with 5.6-kb-long scDNA and the indicated proteins. A representative experiment is shown. The reaction products were separated by electrophoresis in the presence of GelRed that leads to the supercoiling of covalently-closed DNA. Source data are provided as a Source Data file.

Supplementary Figure 2

a

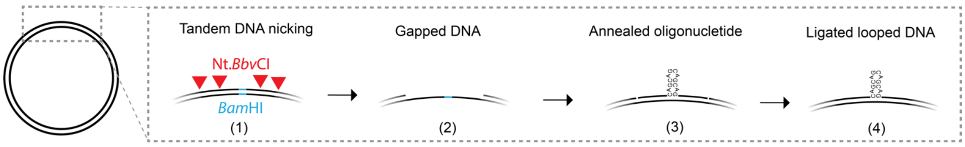

b

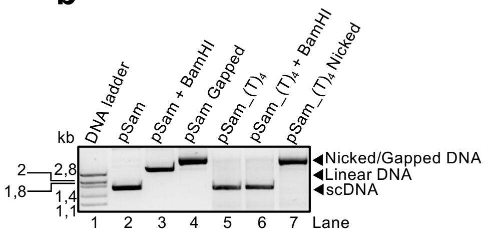

Supplementary Figure 2. Preparation of DNA substrates. a, A schematic of DNA substrate preparation. DNA is first nicked with Nt.BbvCI at adjacent positions (1). The nicked fragments are denatured resulting in Gapped DNA (2). An oligonucleotide with the desired sequence is annealed to the gapped region, giving rise to the extrahelical loop (3). Unligated DNA is enzymatically removed using T5 exonuclease and ligated DNA is purified (4). The desired DNA substrate lacks the BamHI recognition site. **b,** Agarose gel showing the various intermediates of DNA substrate preparation. Lane 2, unmodified pSam. Lane 3, pSam linearized with BamHI. Lane 4, Gapped DNA. Lane 5, ligated and purified pSam_$(T)_4$ substrate. Lane 6, pSam_ $(T)_4$ reacted with BamHI, showing that it is refractory to DNA cleavage. Lane 7, Nicked modified pSam_ $(T)_4$. Source data are provided as a Source Data file.

Supplementary Figure 3

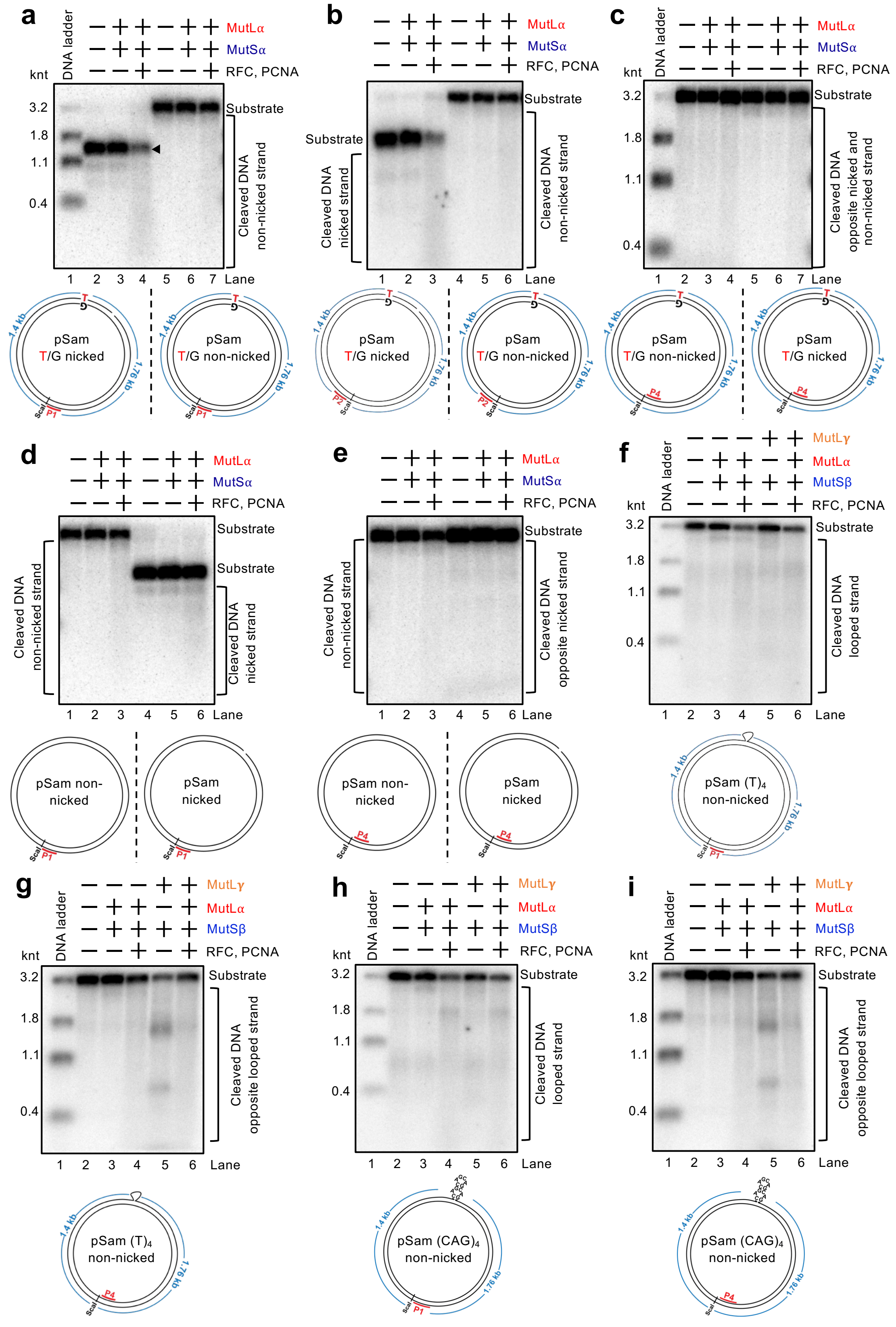

Supplementary Figure 3. Nuclease assays with MutSα and MutLα. Legend on next page.

Supplementary Figure 3. Nuclease assays with MutSα and MutLα. a-c, Nuclease assays with pSam_T/G heteroduplex DNA with or without a nick 3' of the mismatch and the indicated proteins. The reaction products were analyzed by Southern blotting with probes complementary to the nicked strand at various positions, P1 (**a**) or P2 (**b**), or to the strand opposite the nick, P4 (**c**). Top, representative Southern blots. Bottom, cartoons of the DNA substrates. The black triangle in (**a**) indicates the pSam_T/G nicked substrate. **d,e,** Nuclease assays with homoduplex DNA (i.e., no mismatch) with or without a nick and the indicated proteins. The products were analyzed by Southern blotting with probes complementary to the nicked strand P1 (**d**) or to the strand opposite to the nick, P4 (**e**). Top, representative Southern blots. Bottom, cartoons of the DNA substrates. **f,g** Nuclease assays with pSam_$(T)_4$ heteroduplex DNA and the indicated proteins. The reaction products were analyzed by Southern blotting with probes complementary to the looped DNA strand, P1 (**f**), or to the strand opposite the loop, P4 (**g**). Random loading of PCNA on the loop by RFC triggers cleavage by MutLα on both strands (lane 4 vs lane2 in (**f**) and in (**g**)) whereas MutL**γ** targets primarily the strand opposite the loop. **h,i** Nuclease assays with pSam_$(CAG)_4$ heteroduplex DNA and the indicated proteins. The reaction products were analyzed by Southern blotting with probes complementary to the looped DNA strand, P1 (**g**), or to the strand opposite the loop, P4 (**i**). As in case of pSam_$(T)_4$, MutLα cleaves both strands, whereas MutL**γ** targets primarily the strand opposite the loop. When combined, MutLα is the predominant nuclease (compare lane 5 and lane 6 in (**g**) and (**i**)). Source data are provided as a Source Data file.

Supplementary Figure 4

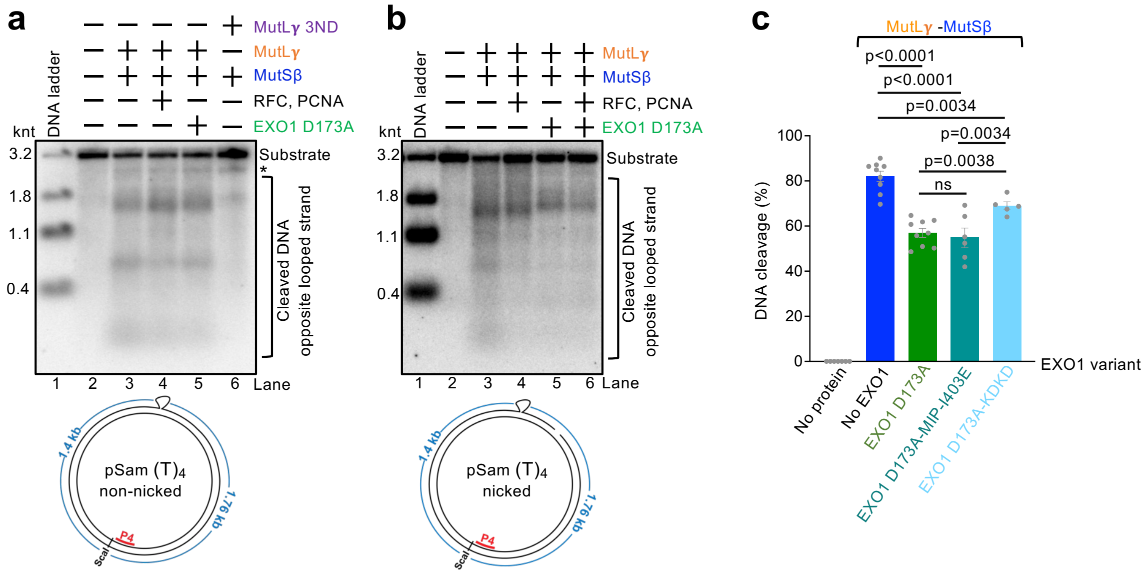

Supplementary Figure 4. Nuclease assays with MutSβ and MutLγ. a,b, Representative nuclease assays with pSam_(T)$_4$ heteroduplex DNA without a nick (**a**) or with a nick 3' of the loop (**b**) and the indicated proteins. The reaction products were analyzed by Southern blotting with a probe complementary to the strand opposite the loop, P4. MutLγ 3ND, nuclease-deficient MutLγ. EXO1 D173A, nuclease-deficient EXO1. *, DNA not cleaved by ScaI. **c**, Quantification of nicking assays performed with 5.6-kb-long supercoiled DNA (scDNA) and the indicated proteins. Averages shown; error bars, s.e.m.; n=9 independent experiments except for EXO1 D173A-MIP-I403E (n=6) and for EXO1 D173A-KDKD (n=5). EXO1 D173A-MIP-I403E contains the point mutations D173A, I403E, F506A, F507A. EXO1 D173A-KDKD contains the point mutations D173A, K185A, K237D. All EXO1 variants were used at 150 nM. Statistical analysis was performed by ordinary one-way ANOVA with Tukey's multiple-comparisons test. ns, non-significant. Source data are provided as a Source Data file.

Supplementary Figure 5

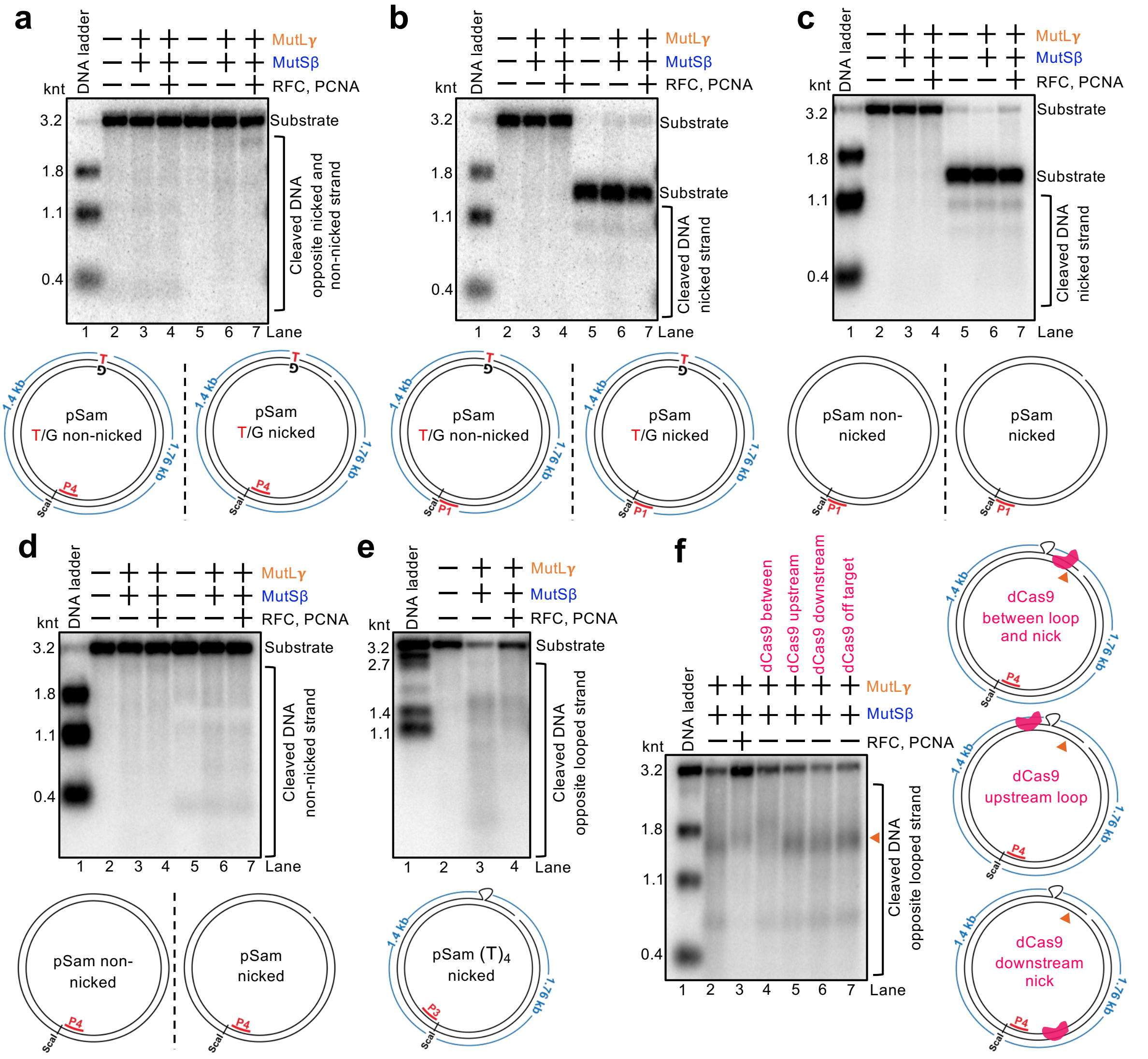

Supplementary Figure 5. Nuclease assays with MutSβ and MutLγ. a,**b**, Representative nuclease assays with pSam_T/G heteroduplex DNA without or with a nick, and the indicated proteins. The reaction products were analyzed by Southern blotting with a probe complementary to the strand opposite the nick, P4 (**a**), or to the nicked strand, P1 (**b**). No apparent DNA cleavage was observed. **c**,**d**, Representative nuclease assays with homoduplex DNA without or with a nick and the indicated proteins. The reaction products were analyzed by Southern blotting with a probe complementary to the nicked strand, P1 (**c**), or opposite the nicked strand, P4 (**d**). No apparent DNA cleavage was observed. **e**, Nuclease assays with pSam_(T)$_4$ heteroduplex DNA with a nick 3' of the loop and the indicated proteins. The reaction products were analyzed by Southern blotting with a probe complementary to the strand opposite the loop, P3. **f**, Nuclease assays with pSam_(T)$_4$ heteroduplex DNA with a nick 3' of the loop, bound by dCas9 as a protein block at various indicated locations, reacted with the indicated proteins. dCas9, catalytically-dead Cas9. The reaction products were analyzed by Southern blotting with a probe complementary to the strand opposite the loop, P4. Left, representative assays; right, cartoons of the DNA substrates with dCas9 at various positions. DNA cleavage at the main MutSβ and MutLγ-dependent DNA incision point, indicated by the orange triangle, was inhibited by RFC, PCNA (lane 3 vs. lane 2) and by dCas9 when placed on the right side of the loop (lane 4 vs. lane 2). The experiments suggest that MutLγ slides primarily in one direction away from the loop, and it is blocked by RFC, PCNA or dCas9. Source data are provided as a Source Data file.

Supplementary Figure 6

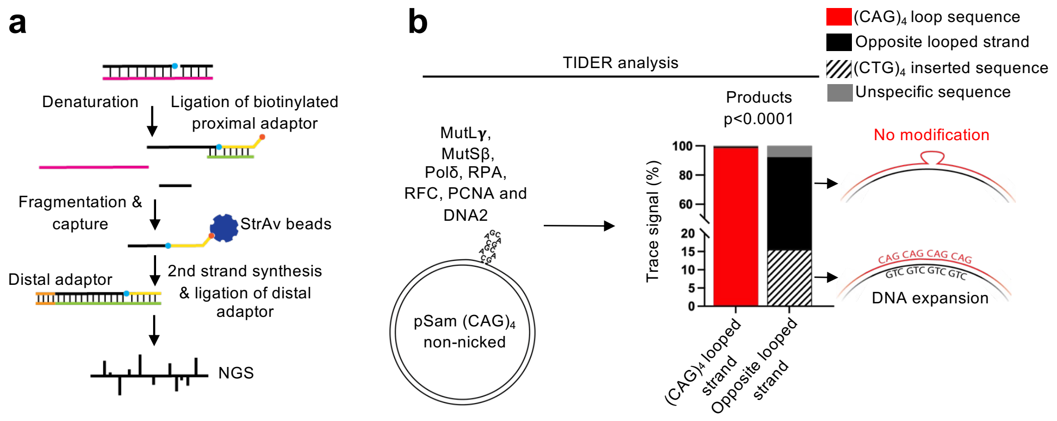

Supplementary Figure 6. A cartoon of the GLOE-seq assay performed in this study and TIDER. a, See Methods for details. **b**, pSam_$(CAG)_4$ DNA was reacted with the indicated proteins, followed by Sanger and TIDER analysis of the reaction products. Same experiment as in Fig. 3c, but with DNA2. By addition of the flap-removing enzyme DNA2, $(CTG)_4$ insertion increased to 15.5%. The *p*-value associated with the trace signal is computed using a two-tailed *t*-test, with the standard errors derived from the variance-covariance matrix. Source data are provided as a Source Data file.

Supplementary Figure 7

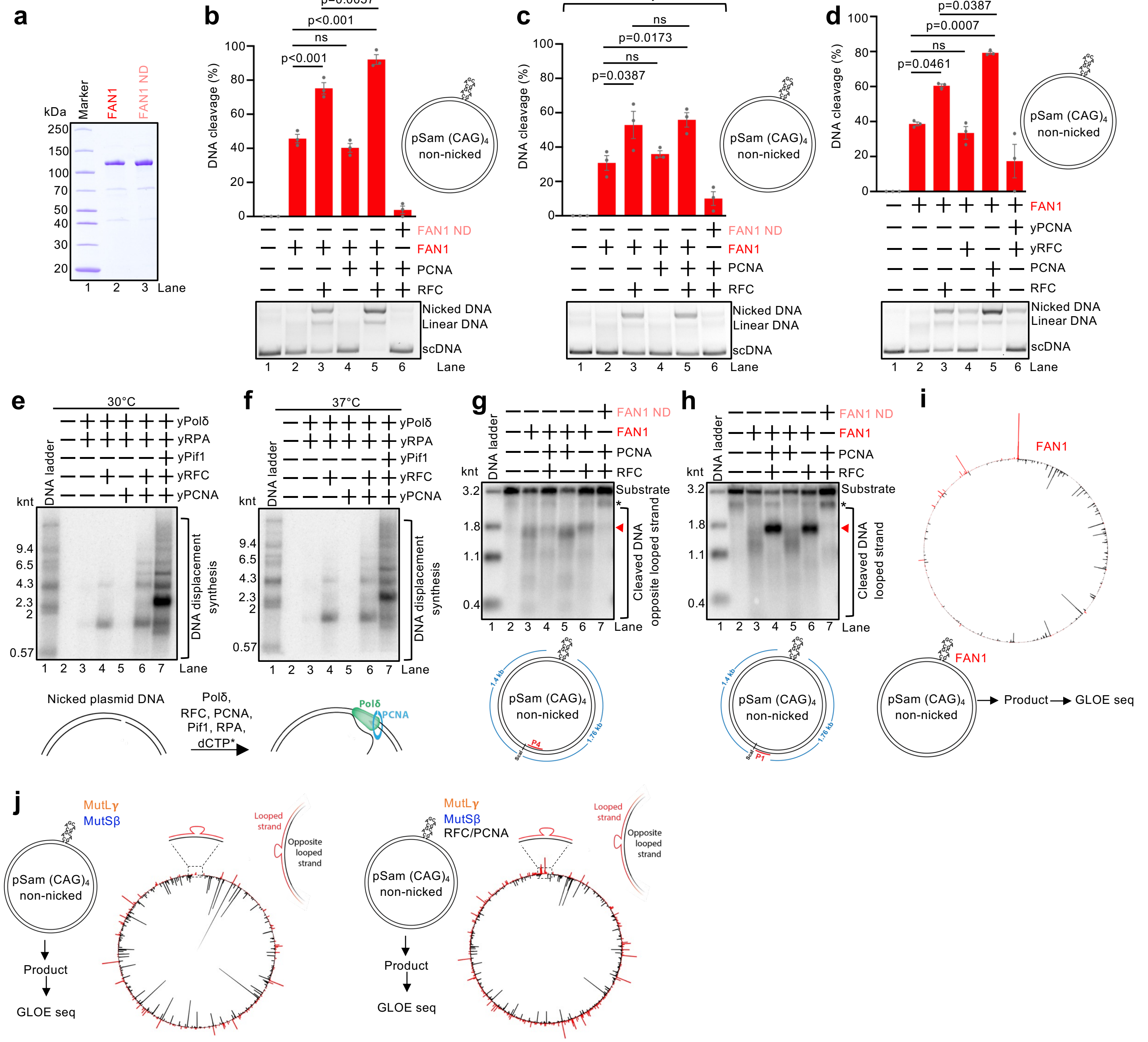

Supplementary Figure 7. Activity of FAN1 on looped DNA substrates and its regulation by RFC and PCNA. a, Recombinant human FAN1 and FAN1 ND used in this study. Nuclease-deficient FAN1 ND contains D960A, D981A, R982A point mutations in the nuclease active site. The polyacrylamide gel was stained with Coomassie Brillant blue. **b**, Representative nicking assays with pSam_(CAG)$_4$ DNA. The DNA substrate was prepared in its relaxed form, and reacted with the indicated proteins. The reaction products were separated by electrophoresis in the presence of GelRed that leads to the supercoiling of covalently-closed DNA. Averages shown; error bars, s.e.m.; n=3 independent experiments. Statistical analysis was performed by ordinary one-way ANOVA with Tukey's multiple-comparisons test. **c**, Representative assay as in (**b**), with non-hydrolysable ATPγS instead of ATP. PCNA has no effect when ATP is not hydrolyzable. Averages shown; error bars, s.e.m.; n=3 independent experiments. Statistical analysis was performed by ordinary one-way ANOVA with Tukey's multiple-comparisons test. ns, non-significant. **d**, Representative nicking assay with pSam_(CAG)$_4$ DNA and the indicated yeast *S. cerevisiae* (y) or human RFC and/or PCNA proteins as indicated. FAN1 is controlled by cognate human RFC and PCNA. Averages shown; error bars, s.e.m.; n=3 independent experiments. Statistical analysis was performed by ordinary one-way ANOVA with Tukey's multiple-comparisons test. **e**,**f**, Top, representative DNA displacement synthesis assay with the indicated proteins at 30 °C (**e**) and 37 °C (**f**). Yeast proteins remain equally active in both conditions. Bottom, a cartoon representative of the assay. **g**,**h**, Representative nuclease assays with pSam_(CAG)$_4$ DNA reacted with the indicated proteins. The reaction products were analyzed by Southern blotting with probes complementary to the strand opposite the loop, P4 (**g**), or to the looped strand, P1 (**h**). The assays show that RFC and PCNA restrict FAN1 incisions of the non-looped strand, while they stimulate incisions of the looped DNA strand (red triangle). *, DNA not cleaved by ScaI. **i**, Polar plot of reads from GLOE-seq assay performed on pSam_(CAG)$_4$ DNA reacted with FAN1 without RFC and PCNA. Compare with Fig. 4e. **j**, Polar plot of reads from GLOE-seq assay performed on pSam_(CAG)$_4$ DNA reacted with MutSβ-MutLγ alone (left) or with RFC and PCNA (right). Source data are provided as a Source Data file.

Supplementary Figure 8

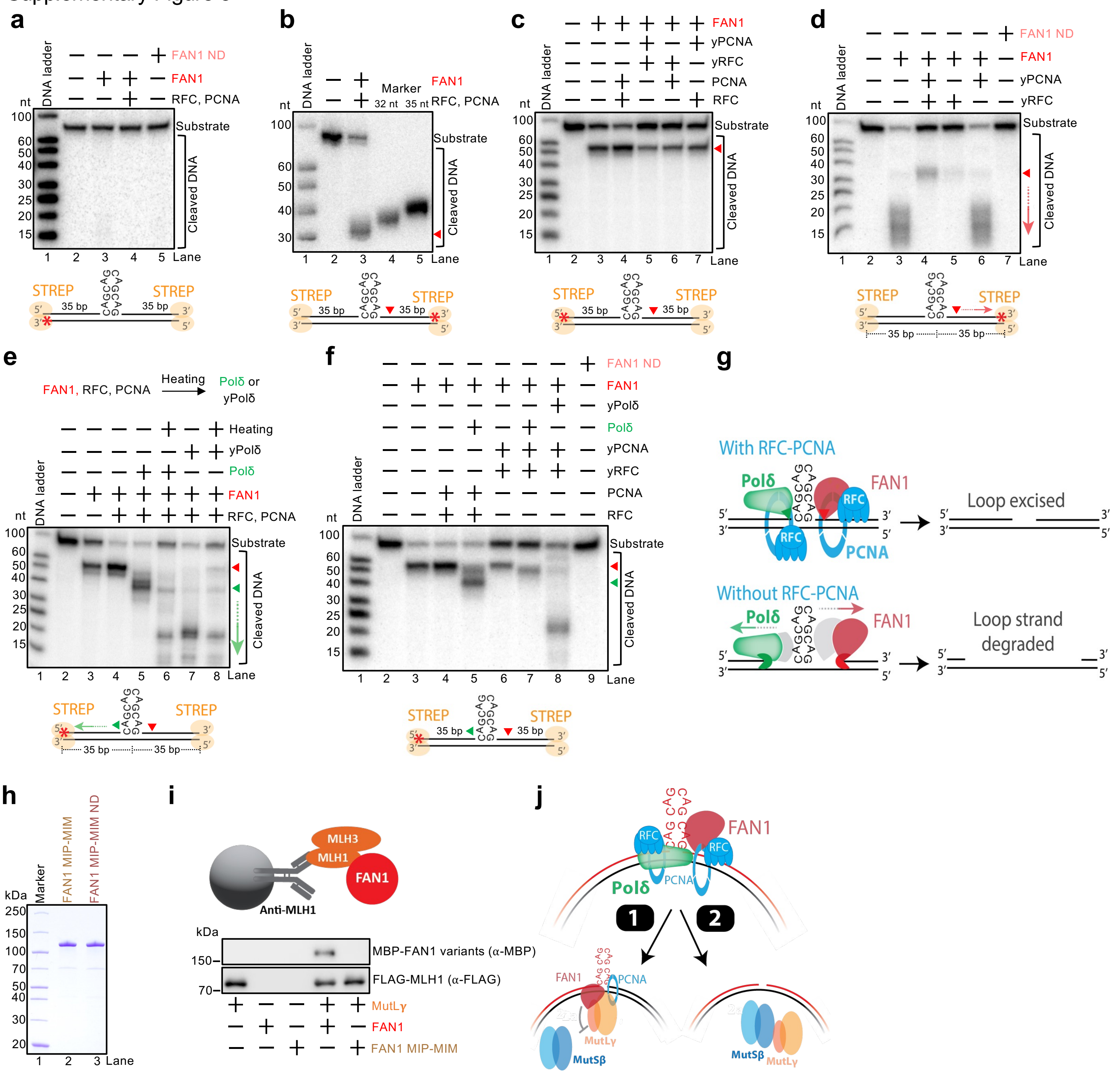

Supplementary Figure 8. Effect of RFC, PCNA and Polδ on the FAN1 nuclease. Legend on next page.

Supplementary Figure 8. Effect of RFC, PCNA and Polδ on the FAN1 nuclease. a-d, Oligonucleotide-based nuclease assays with the indicated proteins and DNA substrates. FAN1 ND, nuclease-deficient FAN1. Reaction products were analyzed by denaturing polyacrylamide gel electrophoresis. Top, representative assays. Bottom, cartoons of the respective DNA substrates. DNA ends were blocked by monovalent streptavidin (STREP). The red asterisk (*) indicates the position of the radioactive label. The red triangle indicates the position of the endonucleolytic DNA incision by FAN1. The red arrow indicates subsequent exonucleolytic DNA degradation by FAN1. In (**a**) the bottom, non-looped DNA strand was 3′-labeled, and no incision by FAN1 was detected. In (**b**) the looped DNA strand was 3′-labeled, and lanes 4 and 5 included markers to monitor FAN1-mediated nuclease activity. In (**c**) the looped DNA strand was 5′-labeled, in (**d**) the looped DNA strand was 3′-labeled and nuclease assays were performed with the indicated proteins to evaluate the individual effects of yeast RFC and yeast PCNA. Unlike the human counterparts, the yeast homologues do not regulate FAN1 activity. **e**,**f**, Oligonucleotide-based nuclease assays with the indicated proteins, analyzed by denaturing polyacrylamide gel electrophoresis. FAN1 ND, nuclease-deficient FAN1 (D960A, D981A, R982A). Top, representative assays. Bottom, cartoons of the respective DNA substrates. DNA ends were blocked by monovalent streptavidin (STREP); the red asterisk (*) indicates the position of the radioactive label. The red and green triangles indicate the position of the DNA incision done by FAN1 or Polδ, respectively. The green arrow indicates subsequent exonucleolytic DNA degradation by Polδ. In (**e**), after incubation with RFC, PCNA, and FAN1, the reaction was either heat-inactivated or left untreated prior to adding either yeast or human Polδ. Human RFC and PCNA could restrict the exonuclease activity of human Polδ (lanes 5 and 6). In contrast, yeast Polδ was not affected by human RFC and PCNA under neither condition. The red triangle indicates the endonucleolytic DNA incisions by FAN1 and the green triangle indicates the removal of the loop by Polδ. The green arrow denotes subsequent exonucleolytic DNA degradation by Polδ, which is restricted by RFC and PCNA. In (**f**), analogous assays to (**e**) were performed using yeast Polδ in the presence of yeast RFC and yeast PCNA. Yeast Polδ was not affected by the yeast components, and yeast RFC and yeast PCNA did not stimulate human Polδ to the same extent as their human counterparts. **g**, A cartoon showing that RFC and PCNA restrict the exonuclease activities of FAN1 and Polδ to limit DNA excision to the looped DNA strand. With RFC and PCNA (top) only the loop is excised. Without RFC and PCNA (bottom) the looped strand is further degraded. **h**, Recombinant human FAN1 MIP-MIM (Y128A, F129A, L155A, L159A) and nuclease-deficient FAN1 MIP-MIM ND (Y128A, F129A, L155A, L159A, D906A, D981A, R982A) used in this study. The polyacrylamide gel was stained with Coomassie Brillant blue. **i**, Protein interaction assay. MutLγ (MLH1-MLH3) was immobilized using anti-MLH1 antibody, followed by incubation with FAN1 or FAN1 MIP-MIM. Top, a cartoon of the assay. Bottom, representative Western blot analysis. **j**, A cartoon showing that FAN1 inhibits MutLγ through both structural (1) and catalytic (2) functions. Source data are provided as a Source Data file.

Supplementary Table 1. List of oligonucleotides used in this study for site-directed mutagenesis and cloning.

Name	Sequence (5' to 3')
FAN1 FW D981A/R982A	GTTAAAGGCCCCAATGCTGCTCTTTCACATAAGCAG
FAN1 REV D981A/R982A	CTGCTTATGTGAAAGAGCAGCATTGGGGCCTTTAAC
FAN1 MIP FW	AAGCAGAAGATCAGTCCCGCCGCTAAAAGTAATGATGTGGTG
FAN1 MIP REV	CACCACATCATTACTTTTAGCGGCGGGACTGATCTTCTGCTT
FAN1 MIM FW	GTCATTTGTTTGGGAAGCGCAGCATCTAAAGCGTCCAGAAAATACGTAAAG
FAN1 MIM REV	CTTTACGTATTTTCTGGACGCTTTAGATGCTGCGCTTCCCAAACAAATGAC
Fw-MBP-yPif1-His-insect	GGTTACGCTAGCCATATGAGTTCAAGAGGTTTCAGATCT
Rv-MBP-yPif1-His-insect	TCACACTTCACGCGTCAGTGGTGGTGCTGATGATGGTGATGATGGTGTTCTAA AATGTGGTCTTCGGT

Supplementary Table 2. List of oligonucleotides used to generate pSam plasmid.

Name	Sequence (5' to 3')
Sam20	GGCCGCGATCTGATCAGATCCACCTCAGCACTTCCTCAGCGAGCCTCAGCGGATC CTCAGCTACCCTCAGCCTGACCTCAGCGTCACCTCAGC
Sam21	AGCTGCTGAGGTGACGCTGAGGTCAGGCTGAGGGTAGCTGAGGATCCGCTGAGG CTCGCTGAGGAAGTGCTGAGGTGGATCTGATCAGATCGC

Supplementary Table 3. pSam plasmid variant sequences.

The standard pSam sequence is listed in black. For pSam_Red and pSam_Blue variants, the inserted sequences have been highlighted with the matching color.

Name	Sequence (5' to 3')
pSam	CCACCTCAGCACTTCCTCAGCGAGCCTCAGCGGATCCTCAGCTACCCTCAG CCTGACCTCAGCGTCACCTCAGCAGCTTGAGTATTCTATAGTGTCACCTAAAT AGCTTGGCGTAATCATGGTCATAGCTGTTTCCTGTGTGAAATTGTTATCCGCT CACAATTCCACACAACATACGAGCCGGAAGCATAAAGTGTAAAGCCTGGGGT GCCTAATGAGTGAGCTAACTCACATTAATTGCGTTGCGCTCACTGCCCGCTTT CCAGTCGGGAAACCTGTCGTGCCAGCTGCATTAATGAATCTGCCAACGCGC GGGGAGAGGCGGTTTGCGTATTGGGCGCTCTTCCGCTTCCTCGCTCACTGA GTCGCTGCGCTCGGTCGTTCGGCTGCGGCGAGCGGTATCAGCTCACTCAAA GGCGGTAATACGGTTATCCACAGAATCAGGGGATAACGCAGGAAAGAACATG TGAGCAAAAGGCCAGCAAAAGGCCAGGAACCGTAAAAAGGCCGCGTTGCTG GCGTTTTTCCATAGGCTCCGCCCCCCTGACGAGCATCACAAAAATCGACGCT

	CAAGTCAGAGGTGGCGAAACCCGACAGGACTATAAAGATACCAGGCGTTTCC<br>CCCTGGAAGCTCCCTCGTGCGCTCTCCTGTTCCGACCCTGCCGCTTACCGG<br>ATACCTGTCCGCCTTTCTCCCTTCGGGAAGCGTGGCGCTTTCTCATAGCTCA<br>CGCTGTAGGTATCTCAGTTCGGTGTAGGTCGTTCGCTCCAAGCTGGGCTGTG<br>TGCACGAACCCCCCGTTCAGCCCGACCGCTGCGCCTTATCCGGTAACTATCG<br>TCTTGAATCCAACCCGGTAAGACACGACTTATCGCCACTGGCAGCAGCCACT<br>GGTAACAGGATTAGCAGAGCGAGGTATGTAGGCGGTGCTACAGAGTTCTTGA<br>AGTGGTGGCCTAACTACGGCTACACTAGAAGAACAGTATTTGGTATCTGCGCT<br>CTGCTGAAGCCAGTTACCTTCTGAAAAAGAGTTGGTAGCTCTTGATCCGGCA<br>AACAAACCACCGCTGGTAGCGGTGGTTTTTTTGTTTGCAAGCAGCAGATTAC<br>GCGCAGAAAAAAAGGATCTCAAGAAGATCCTTTGATCTTTTCTACGGGGTCT<br>GACGCTCAGTGGAACGAAAACTCACGTTAAGGGATTTTGGTCATGAGATTATC<br>AAAAAGGATCTTCACCTAGATCCTTTTAAATTAAAAATGAAGTTTTAAATCAATC<br>TAAAGTATATATGAGTAAACTTGGTCTGACAGTTACCAATGCTTAATCAGTGAG<br>GCACCTATCTCAGCGATCTGTCTATTTCGTTCATCCATAGTTGCCTGGCTCCC<br>CGTCGTGTAGATAACTACGATACGGGAGGGCTTACCATCTGGCCCCAGTGCT<br>GCAATGATACCGCGAGACCCACGCTCACCGGCTCCAGATTTATCAGCAATAA<br>ACCAGCCAGCCGGAAGGGCCGAGCGCAGAAGTGGTCCTGCAACTTTATCCG<br>CCTCCATCCAGTCTATTAATTGTTGCCGGGAAGCTAGAGTAAGTAGTTCGCCA<br>GTTAATAGTTTGCGCAACGTTGTTGCCATTGCTACAGGCATCGTGGTGTCACG<br>CTCGTCGTTTGGTATGGCTTCATTCAGCTCCGGTTCCCAACGATCAAGGCGA<br>GTTACATGATCCCCCATGTTGTGCAAAAAAGCGGTTAGCTCCTTCGGTCCTC<br>CGATCGTTGTCAGAAGTAAGTTGGCCGCAGTGTTATCACTCATGGTTATGGCA<br>GCACTGCATAATTCTCTTACTGTCATGCCATCCGTAAGATGCTTTTCTGTGACT<br>GGTGAGTACTCAACCAAGTCATTCTGAGAATAGTGTATGCGGCGACCGAGTT<br>GCTCTTGCCCGGCGTCAATACGGGATAATACCGCGCCACATAGCAGAACTTT<br>AAAAGTGCTCATCATTGGAAAACGTTCTTCGGGGCGAAAACTCTCAAGGATC<br>TTACCGCTGTTGAGATCCAGTTCGATGTAACCCACTCGTGCACCCAACTGAT<br>CTTCAGCATCTTTTACTTTCACCAGCGTTTCTGGGTGAGCAAAAACAGGAAG<br>GCAAAATGCCGCAAAAAAGGGAATAAGGGCGACACGGAAATGTTGAATACTC<br>ATACTCTTCCTTTTTCAATATTATTGAAGCATTTATCAGGGTTATTGTCTCATGA<br>GCGGATACATATTTGAATGTATTTAGAAAAATAAACAAATAGGGGTTCCGCGCA<br>CATTTCCCCGAAAAGTGCCACCTGACGTCTAAGAAACCATTATTATCATGACAT<br>TAACCTATAAAAATAGGCGTATCACGAGGCCCTTTCGTCTCGCGCGTTTCGGT<br>GATGACGGTGAAAACCTCTGACACATGCAGCTCCCGGAGACGGTCACAGCT<br>TGTCTGTAAGCGGATGCCGGGAGCAGACAAGCCCGTCAGGGCGCGTCAGC<br>GGGTGTTGGCGGGTGTCGGGGCTGGCTTAACTATGCGGCATCAGAGCAGAT<br>TGTACTTAGAGTGCACCATATGCGGTGTGAAATACCGCACAGATGCGTAAGGA<br>GAAAATACCGCATCAGGAAATTGTAAGCGTTAATATTTTGTTAAAATTCGCGTT<br>AAATTTTTGTTAAATCAGCTCATTTTTTAACCAATAGGCCGAAATCGGCAAAAT

	CCCTTATAAATCAAAAGAATAGACCGAGATAGGGTTGAGTGTTGTTCCAGTTT<br>GGAACAAGAATCCACTATTAAAGAACGTGGAATCCAACGTCAAAGGGCGAAA<br>AACCGTCTATCAGGGCGATGGCCCACTACGTGAACCATCACCCTAATCAAGT<br>TTTTTGGGGTCGAGGTGCCGTAAAGCACTAAATCGGAACCCTAAAGGGAGCC<br>CCCGATTTAGAGCTTGACGGGGAAAGCCGGCGAACGTGGCGAGAAAGGAA<br>GGGAAGAAAGCGAAAGTAGCGGGCGCTAGGGCGCTGGCAAGTGTAGCGGT<br>CACGCTGCGCGTAACCACCACACCCGCCGCGCTTAATGCGCCGCTACAGGG<br>CGCGTCCATTCGCCATTCAGGCTGCGCAACTGTTGGGAAGGGCGATCGGTG<br>CGGGCCTCTTCGCTATTACGCCAGCTGGCGAAAGGGGGATGTGCTGCAAGG<br>CGATTAAGTTGGGTAACGCCAGGGTTTTCCCAGTCACGACGTTGTAAAACGA<br>CGGCCAGTGAATTGTAATACGAACACTATAGGGCGAATTGGCGGCCGCGATC<br>TGATCAGAT
pSam_Red	CCACCTCAGCACTTCCTCAGCGAGCCTCAGCGGATCCTCAGCTACCCTCAG<br>CCTGACCTCAGCGTCACCTCAGCAGCTTGAGTATTCTATAGTGTCACCTAAAT<br>AGCTTGGCGTAATCATGGTCATAGCTGTTTCCTGTGTGAAATTGTTATCCGCT<br>CACAATTCCACACAACATACGAGCCGGAAGCATAAAGTGTAAAGCCTGGGGT<br>GCCTAATGAGTGAGCTAACTCACATTAATTGCGTTGCGCTCACTGCCCGCTTT<br>CCAGTCGGGAAACCTGTCGTGCCAGCTGCATTAATGAATCTGCCAACGCGC<br>GGGGAGAGGCGGTTTGCGTATTGGGCGCTCTTCCGCTTCCTCGCTCACTGA<br>GTCGCTGCGCTCGGTCGTTCGGCTGCGGCGAGCGGTATCAGCTCACTCAAA<br>GGCGGTAATACGGTTATCCACAGAATCAGGGGATAACGCAGGAAAGAACATG<br>TGAGCAAAAGGCCAGCAAAAGGCCAGGAACCGTAAAAAGGCCGCGTTGCTG<br>GCGTTTTTCCATAGGCTCCGCCCCCCTGACGAGCATCACAAAAATCGACGCT<br>CAAGTCAGAGGTGGCGAAACCCGACAGGACTATAAAGATACCAGGCGTTTCC<br>CCCTGGAAGCTCCCTCGTGCGCTCTCCTGTTCCGACCCTGCCGCTTACCGG<br>ATACCTGTCCGCCTTTCTCCCTTCGGGAAGCGTGGCGCTTTCTCATAGCTCA<br>CGCTGTAGGTATCTCAGTTCGGTGTAGGTCGTTCGCTCCAAGCTGGGCTGTG<br>TGCACGAACCCCCCGTTCAGCCCGACCGCTGCGCCTTATCCGGTAACTATCG<br>TCTTGAATCCAACCCGGTAAGACACGACTTATCGCCACTGGCAGCAGCCACT<br>GGTAACAGGATTAGCAGAGCGAGGTATGTAGGCGGTGCTACAGAGTTCTTGA<br>AGTGGTGGCCTAACTACGGCTACACTAGAAGAACAGTATTTGGTATCTGCGCT<br>CTGCTGAAGCCAGTTACCTTCTGAAAAAGAGTTGGTAGCTCTTGATCCGGCA<br>AACAAACCACCGCTGGTAGCGGTGGTTTTTTTGTTTGCAAGCAGCAGATTAC<br>GCGCAGAAAAAAAGGATCTCAAGAAGATCCTTCTGAAAAAAGAGTTGGTAGC<br>TCTTGATCCGGCAAACAAACCACCGCTGGTAGCGGTGGTTTTTTTGTTTGCA<br>AGCAGCAGATTACGCGCAGAAAAAAAGGATCTCAAGAAGATCCTTTGATCTTT<br>TCTACGGGGTCTGACGCTCAGTGGAACGAAAACTCACGTTAAGGGATTTTGG<br>TCATGAGATTATCAAAAAGGATCTTCACCTAGATCCTTTTAAATTAAAAATGAAG<br>TTTTAAATCAATCTAAAGTATATATGAGTAAACTTGGTCTGACAGTTACCAATGC<br>TTAATCAGTGAGGCACCTATCTCAGCGATCTGTCTATTTCGTTCATCCATAGTT

	GCCTGGCTCCCCGTCGTGTAGATAACTACGATACGGGAGGGCTTACCATCTG GCCCCAGTGCTGCAATGATACCGCGAGACCCACGCTCACCGGCTCCAGATT TATCAGCAATAAACCAGCCAGCCGGAAGGGCCGAGCGCAGAAGTGGTCCTG CAACTTTATCCGCCTCCATCCAGTCTATTAATTGTTGCCGGGAAGCTAGAGTA AGTAGTTCGCCAGTTAATAGTTTGCGCAACGTTGTTGCCATTGCTACAGGCAT CGTGGTGTCACGCTCGTCGTTTGGTATGGCTTCATTCAGCTCCGGTTCCCAA CGATCAAGGCGAGTTACATGATCCCCCATGTTGTGCAAAAAAGCGGTTAGCT CCTTCGGTCCTCCGATCGTTGTCAGAAGTAAGTTGGCCGCAGTGTTATCACT CATGGTTATGGCAGCACTGCATAATTCTCTTACTGTCATGCCATCCGTAAGATG CTTTTCTGTGACTGGTGAGTACTCAACCAAGTCATTCTGAGAATAGTGTATGC GGCGACCGAGTTGCTCTTGCCCGGCGTCAATACGGGATAATACCGCGCCAC ATAGCAGAACTTTAAAAGTGCTCATCATTGGAAAACGTTCTTCGGGGCGAAAA CTCTCAAGGATCTTACCGCTGTTGAGATCCAGTTCGATGTAACCCACTCGTG CACCCAACTGATCTTCAGCATCTTTTACTTTCACCAGCGTTTCTGGGTGAGCA AAAACAGGAAGGCAAAATGCCGCAAAAAAGGGAATAAGGGCGACACGGAAA TGTTGAATACTCATACTCTTCCTTTTTCAATATTATTGAAGCATTTATCAGGGTT ATTGTCTCATGAGCGGATACATATTTGAATGTATTTAGAAAAATAAACAAATAGG GGTTCCGCGCACATTTCCCCGAAAAGTGCCACCTGACGTCGCTCGTGCTGA GCGTACCGTTATGCGCCAGGACAACAGGGATACCGTCGACGATACTGTTTCG TCCGAGTCATTGCAATCGCTGTTCTCCGAATGGGATAACCCCGTGTTCGCCC GTTACCCTGAGGTGGCTGTCGACGTTTCTAGCGGACAGGCCGAATCCCTCG CTGTCAAGATCCACAACATCTTGTACCCTTACCGCTTCACCAAGGGTATGATC CACTCTATGCAAGTGCTCCAGCAAGTCGACAACAAGTTCATCGCTTGCTTGAT GAGCACTAAGACAGAGGAAAACGGCGAGGCCGGAGGTAACCTGCTCGTGCT GGTCGATCAGCACGCTGCCCACGAGCGTATCCGCCTCGAACAATTGATCATC GACTCCTACGAGAAGCAGCAAGCTCAGGGATCTGGTCGCAAGAAGTTGCTG TCATCGACCTTGATCCCCCCTCTGGAAATCACCGTGACTGAGGAACAACGTC GCCTCTTGTGGTGCTACCACAAGAACCTGGAGGACCTCGGATTGGAATTCGT CTTCCCAGACACTTCGGATTCCCTGGTTCTCGTGGGCAAGGTGCCGTTGTGT TTCGTCGAGAGAGAAGCCAACGAGCTGAGGAGAGGCCGTTCAACAGTTACC AAGTCGATCGTGGAGGAATTCATCCGTGAGCAGCTCGAACTGCTCCAAACTA CAGGCGGAATCCAGGGAACATTGCCCCTGACCGTTCAGAAGGTGCTGGCCT CTCAAGCTTGCCACGGTGCTATCAAGTTCAACGACGGCCTCTCGTTGCAAGA GTCCTGTAGACTCATCGAAGCCTTGCGGCCGCGATCTGATCAGAT
pSam_Blue	CCACCTCAGCACTTCCTCAGCGAGCCTCAGCGGATCCTCAGCTACCCTCAG CCTGACCTCAGCGTCACCTCAGCAGCTTGAGTATTCTATAGTGTCACCTAAAT AGCTTGGCGTAATCATGGTCATAGCTGTTTCCTGTGTGAAATTGTTATCCGCT CACAATTCCACACAACATACGAGCCGGAAGCATAAAGTGTAAAGCCTGGGGT GCCTAATGAGTGAGCTAACTCACATTAATTGCGTTGCGCTCACTGCCCGCTTT CCAGTCGGGAAACCTGTCGTGCCAGCTGCATTAATGAATCTGCCAACGCGC

	GGGGAGAGGCGGTTTGCGTATTGGGCGCTCTTCCGCTTCCTCGCTCACTGA<br>GTCGCTGCGCTCGGTCGTTCGGCTGCGGCGAGCGGTATCAGCTCACTCAAA<br>GGCGGTAATACGGTTATCCACAGAATCAGGGGATAACGCAGGAAAGAACATG<br>TGAGCAAAAGGCCAGCAAAAGGCCAGGAACCGTAAAAAGGCCGCGTTGCTG<br>GCGTTTTTCCATAGGCTCCGCCCCCCTGACGAGCATCACAAAAATCGACGCT<br>CAAGTCAGAGGTGGCGAAACCCGACAGGACTATAAAGATACCAGGCGTTTCC<br>CCCTGGAAGCTCCCTCGTGCGCTCTCCTGTTCCGACCCTGCCGCTTACCGG<br>ATACCTGTCCGCCTTTCTCCCTTCGGGAAGCGTGGCGCTTTCTCATAGCTCA<br>CGCTGTAGGTATCTCAGTTCGGTGTAGGTCGTTCGCTCCAAGCTGGGCTGTG<br>TGCACGAACCCCCCGTTCAGCCCGACCGCTGCGCCTTATCCGGTAACTATCG<br>TCTTGAATCCAACCCGGTAAGACACGACTTATCGCCACTGGCAGCAGCCACT<br>GGTAACAGGATTAGCAGAGCGAGGTATGTAGGCGGTGCTACAGAGTTCTTGA<br>AGTGGTGGCCTAACTACGGCTACACTAGAAGAACAGTATTTGGTATCTGCGCT<br>CTGCTGAAGCCAGTTACCTTCTGAAAAAGAGTTGGTAGCTCTTGATCCGGCA<br>AACAAACCACCGCTGGTAGCGGTGGTTTTTTTGTTTGCAAGCAGCAGATTAC<br>GCGCAGAAAAAAAGGATCTCAAGAAGATCCTTTGATCTTTTCTACGGGGTCT<br>GACGCTCAGTGGAACGAAAACTCACGTTAAGGGATTTTGGTCATGAGATTATC<br>AAAAAGGATCTTCACCTAGATCCTTTTAAATTAAAAATGAAGTTTTAAATCAATC<br>TAAAGTATATATGAGTAAACTTGGTCTGACAGTTACCAATGCTTAATCAGTGAG<br>GCACCTATCTCAGCGATCTGTCTATTTCGTTCATCCATAGTTGCCTGGCTCCC<br>CGTCGTGTAGATAACTACGATACGGGAGGGCTTACCATCTGGCCCCAGTGCT<br>GCAATGATACCGCGAGACCCACGCTCACCGGCTCCAGATTTATCAGCAATAA<br>ACCAGCCAGCCGGAAGGGCCGAGCGCAGAAGTGGTCCTGCAACTTTATCCG<br>CCTCCATCCAGTCTATTAATTGTTGCCGGGAAGCTAGAGTAAGTAGTTCGCCA<br>GTTAATAGTTTGCGCAACGTTGTTGCCATTGCTACAGGCATCGTGGTGTCACG<br>CTCGTCGTTTGGTATGGCTTCATTCAGCTCCGGTTCCCAACGATCAAGGCGA<br>GTTACATGATCCCCCATGTTGTGCAAAAAAGCGGTTAGCTCCTTCGGTCCTC<br>CGATCGTTGTCAGAAGTAAGTTGGCCGCAGTGTTATCACTCATGGTTATGGCA<br>GCACTGCATAATTCTCTTACTGTCATGCCATCCGTAAGATGCTTTTCTGTGACT<br>GGTGAGTACTCAACCAAGTCATTCTGAGAATAGTGTATGCGGCGACCGAGTT<br>GCTCTTGCCCGGCGTCAATACGGGATAATACCGCGCCACATAGCAGAACTTT<br>AAAAGTGCTCATCATTGGAAAACGTTCTTCGGGGCGAAAACTCTCAAGGATC<br>TTACCGCTGTTGAGATCCAGTTCGATGTAACCCACTCGTGCACCCAACTGAT<br>CTTCAGCATCTTTTACTTTCACCAGCGTTTCTGGGTGAGCAAAAACAGGAAG<br>GCAAAATGCCGCAAAAAAGGGAATAAGGGCGACACGGAAATGTTGAATACTC<br>ATACTCTTCCTTTTTCAATATTATTGAAGCATTTATCAGGGTTATTGTCTCATGA<br>GCGGATACATATTTGAATGTATTTAGAAAAATAAACAAATAGGGGTTCCGCGCA<br>CATTTCCCCGAAAAGTGCCACCTGACGTCGGACTGGCCATCTCCTCTCTGG<br>GTCAATGCGTGGAGGAATTGGCTCTGAACAGCATCGATGCTGAGGCCAAGT<br>GTGTCGCCGTTCGCGTGAACATGGAAACCTTCCAGGTCCAAGTTATCGACAA

	CGGTTTCGGCATGGGATCTGACGATGTCGAGAAGGTTGGTAACAGGTACTTC ACTTCTAAGTGCCACAGCGTCCAGGATTTGGAGAACCCTAGATTCTACGGCT TCCGTGGAGAAGCTCTGGCCAACATCGCTGACATGGCTTCCGCCGTGGAGA TCAGCTCAAAGAAGAACCGCACTATGAAGACATTCGTCAAGTTGTTCCAGTCT GGCAAGGCTCTGAAGGCCTGCGAAGCTGACGTGACCCGTGCTTCCGCTGG AACCACTGTGACCGTCTACAACCTCTTCTACCAGTTGCCCGTTCGTCGCAAG TGTATGGACCCTAGACTCGAGTTCGAAAAGGTGCGTCAACGCATCGAGGCTC TCTCCTTGATGCACCCGAGCATCTCATTCTCGCTGCGTAACGATGTTTCCGG CTCTATGGTGCTGCAGCTCCCCAAGACCAAGGACGTGTGCTCCCGTTTCTGT CAGATCTACGGTCTCGGCAAGTCTCAAAAGTTGCGTGAGATCAGCTTCAAGT ACAAGGAGTTCGAATTGAGCGGATACATCTCGTCCGAAGCCCACTACAACAA GAACATGCAGTTCCTGTTCGTCAACAAGAGGTTGGTTCTGAGAACTAAGTTG CACAAGCTGATCGACTTCCTGCTCAGAAAGGAGTCCATCATCTGCAAGCCAA AGAACGGCCCGACCTCTCGTCAGATGAACTCTAGCCTGAGGCACAGAAGCA CTCCCGAGCTCTACGGAATCTACGCGGCCGCGATCTGATCAGAT

Supplementary Table 4. List of oligonucleotides used in this study for substrate preparation. The bold **T**/**G** represent the site of the biotin modification. The sequence of the extrahelical loop or mismatch is indicated in red.

Name	Sequence (5' to 3')
Tloop	TCAGCACTTCCTCAGCGAGCCTCAGCGGATTTTTCCTCAGCTAC CCTCAGCCTGACCTCAGCGTCACC
T/G	TCAGCACTTCCTCAGCGAGCCTCAGCGGATTCTCAGCTACCCTC AGCCTGACCTCAGCGTCACC
Tloop_bottom	TGAGGTGACGCTGAGGTCAGGCTGAGGGTAGCTGAGGATTTTT CCGCTGAGGCTCGCTGAGGAAGTGC
CAG_X4	TCAGCACTTCCTCAGCGAGCCTCAGCGGACAGCAGCAGCAGTC CTCAGCTACCCTCAGCCTGACCTCAGCGTCACC
CAG_X4_top_strand_biotin	ACG**T**CAGAGTCGCTGCGCTCAGTCGTTCAACTGCTCAGCAGCA GCAGTAACTATCGTCTTGAATCCAACCCGGTAAGA**T**ACG
CAG_X4_bottom_strand_biotin	**G**CGTATCTTACCGGGTTGGATTCAAGACGATAGTTAAGCAGTTGA ACGACTGAGCGCAGCGACTCTGACG**T**
Nick_right_part	TAACTATCGTCTTGAATCCAACCCGGTAAGA**T**ACG
Top_strand_3nt_nicked_left_part	ACG**T**CAGAGTCGCTGCGCTCAGTCGTTCAACTGCTCAGCAGCA GCAGTAA
Top strand oligo WT FRP	ACGTCAGAGTCGCTGCGCTCAGTCGTTCAACTGCTTAACTATCG TCTTGAATCCAACCCGGTAAGATACG
Top strand oligo right part WT FRP	TAACTATCGTCTTGAATCCAACCCGGTAAGATACG

Top strand oligo left part WT FRP	ACGTCAGAGTCGCTGCGCTCAGTCGTTCAACTGCT

Supplementary Table 5. List of oligonucleotides used in this study to generate dCas9 RNPs.

Name	Sequence (5' to 3')
Between structure and nick	TTCCACACAACATACGAGCCGTTTTAGAGCTATGCT
Upstream the structure	CTCTTCGCATACGCCAGCGTTTTAGAGCTATGCT
Downstream the nick	AAATCGACGCTCAAGTCAGAGTTTTAGAGCTATGCT
Off target	TACTCGGGAAACATATGAATGTTTTAGAGCTATGCT

Supplementary Table 6. List of oligonucleotides used in this study as probes for Southern blotting.

Name	Sequence (5' to 3')
P1	GGACAGAAAAGCATCTTACGGATGGCATGACA
P2	GGCGGTCGCCGCATACACTATTCTCAGAATGA
P3	GGTCATTCTGAGAATAGTGTATGCGGCGACCG
P4	GGTGTCATGCCATCCGTAAGATGCTTTTCTGT

Supplementary Table 7. List of oligonucleotides used in this study for Sanger sequencing.

Name	Sequence (5' to 3')
Sam13	AACGCAATTAATGTGAGTTAGCTCA
Sam14	GCGTTGGCAGATTCATTAATGCAGC
Sam15	TTCGCCATTCAGGCTGCGCAACTGT
Sam16	CACTATTAAAGAACGTGGAATCCAA
