# Supplementary material for: Mechanism of trinucleotide repeat expansion by MutSβ-MutLγ and contraction by FAN1
Source: Nat Commun. 2025 Oct 27;16:9445. doi: 10.1038/s41467-025-64485-w (PMC12559329; doi:10.1038/s41467-025-64485-w)
Supplement: Supplementary file 2 — Reporting Summary [file 41467_2025_64485_MOESM2_ESM.pdf]

nature portfolio

Corresponding author(s): Petr Cejka

Last updated by author(s): 22 August 2025

Reporting Summary

Nature Portfolio wishes to improve the reproducibility of the work that we publish. This form provides structure for consistency and transparency in reporting. For further information on Nature Portfolio policies, see our Editorial Policies and the Editorial Policy Checklist.

Statistics

For all statistical analyses, confirm that the following items are present in the figure legend, table legend, main text, or Methods section.

n/a	Confirmed	
☐	☒	The exact sample size (n) for each experimental group/condition, given as a discrete number and unit of measurement
☐	☒	A statement on whether measurements were taken from distinct samples or whether the same sample was measured repeatedly
☐	☒	The statistical test(s) used AND whether they are one- or two-sided<br>*Only common tests should be described solely by name; describe more complex techniques in the Methods section.*
☒	☐	A description of all covariates tested
☒	☐	A description of any assumptions or corrections, such as tests of normality and adjustment for multiple comparisons
☐	☒	A full description of the statistical parameters including central tendency (e.g. means) or other basic estimates (e.g. regression coefficient) AND variation (e.g. standard deviation) or associated estimates of uncertainty (e.g. confidence intervals)
☐	☒	For null hypothesis testing, the test statistic (e.g. F, t, r) with confidence intervals, effect sizes, degrees of freedom and P value noted<br>*Give P values as exact values whenever suitable.*
☒	☐	For Bayesian analysis, information on the choice of priors and Markov chain Monte Carlo settings
☒	☐	For hierarchical and complex designs, identification of the appropriate level for tests and full reporting of outcomes
☒	☐	Estimates of effect sizes (e.g. Cohen's d, Pearson's r), indicating how they were calculated

Our web collection on statistics for biologists contains articles on many of the points above.

Software and code

Policy information about availability of computer code

Data collection	We used commercial software, available as a package with the respective instrument, for data collection. Gels were acquired using Typhoon Phosphor Imager FLA 9500, version 1.0, Quantum CX5 Edge 18.06 and photo scanner operated with CanoScan 9000F Mark II scanner and ImageCapture v6.6(525) software. Blots were captured using Fusion FX7 Edge 18.12-5. AcquireMP (Refeyn Ltd, Version AcquireMP 2023 R1.1). GLOE-seq libraries were sequenced using NextSeq 2000.
Data analysis	Data analysis was conducted using only commercially availabe or publicly accessible software. This includes ImageJ2 (NIH, Version 2.9.0/1.53t) for the analysis of gel data; graphs and numerical data (including statistics/error bars) was analyzed and plotted by Prism10 (GraphPad, Version 10.2.3). Custom Python script used to analyze GLOE-seq data is available on GitHub: https://github.com/simonemoro/cutFinder

For manuscripts utilizing custom algorithms or software that are central to the research but not yet described in published literature, software must be made available to editors and reviewers. We strongly encourage code deposition in a community repository (e.g. GitHub). See the Nature Portfolio guidelines for submitting code & software for further information.

Data

Policy information about availability of data

All manuscripts must include a data availability statement. This statement should provide the following information, where applicable:

- Accession codes, unique identifiers, or web links for publicly available datasets
- A description of any restrictions on data availability
- For clinical datasets or third party data, please ensure that the statement adheres to our policy

The code for data processing and analysis of GLOE-seq are available in GitHub https://github.com/simonemoro/cutFinder. The FASTQ files generated from the sequencing of GLOE-seq libraries have been deposited in the European Nucleotide Archive (ENA) at EMBL-EBI (https://www.ebi.ac.uk/ena/browser/home) under the study accession number PRJEB91651. The raw read accession numbers are ERR15195591, ERR15195592, ERR15195593, ERR15195594, ERR15195595 and ERR15195596. Uncropped gel images and numerical source data for graphs are included in the source data file. Protein expression constructs are available on request.

Research involving human participants, their data, or biological material

Policy information about studies with human participants or human data. See also policy information about sex, gender (identity/presentation), and sexual orientation and race, ethnicity and racism.

Reporting on sex and gender	N/A
Reporting on race, ethnicity, or other socially relevant groupings	N/A
Population characteristics	N/A
Recruitment	N/A
Ethics oversight	N/A

Note that full information on the approval of the study protocol must also be provided in the manuscript.

Field-specific reporting

Please select the one below that is the best fit for your research. If you are not sure, read the appropriate sections before making your selection.

☒ Life sciences ☐ Behavioural & social sciences ☐ Ecological, evolutionary & environmental sciences

For a reference copy of the document with all sections, see nature.com/documents/nr-reporting-summary-flat.pdf

Life sciences study design

All studies must disclose on these points even when the disclosure is negative.

Sample size	Sample size (or number of repeats) was chosen based on what is common in the field, and what was practical to do.
Data exclusions	In general, no data were excluded unless there was a valid reason to do so, e.g. experiments with failed positive controls indicating technical problems, or when loading control indicated unequal loading that invalidated the analysis or other technical issues (broken gels, collapsed wells in gels etc.).
Replication	The experiments were repeated multiple times, as indicated in the figure legend. Oftentimes, even within a single experiment, multiple enzyme concentrations were analyzed, or samples were compared at multiple time points. This also contributes to the robustness of the data.
Randomization	Randomization is not relevant to the experiments performed in this study. This study is not sensitive to any biased analysis.
Blinding	Blinding is not relevant to the experiments performed in this study, as measurements were objectively quantified by dedicated software or simply visually presented. Furthermore, the loading order or samples on gels prevented blinding.

Reporting for specific materials, systems and methods

We require information from authors about some types of materials, experimental systems and methods used in many studies. Here, indicate whether each material, system or method listed is relevant to your study. If you are not sure if a list item applies to your research, read the appropriate section before selecting a response.

Materials & experimental systems

n/a	Involved in the study
☐	☒ Antibodies
☐	☒ Eukaryotic cell lines
☒	☐ Palaeontology and archaeology
☒	☐ Animals and other organisms
☒	☐ Clinical data
☒	☐ Dual use research of concern
☒	☐ Plants

Methods

n/a	Involved in the study
☒	☐ ChIP-seq
☒	☐ Flow cytometry
☒	☐ MRI-based neuroimaging

Antibodies

Antibodies used

In the protein interaction assay in Figure 6b and Supplementary Figure 8i we have used the following antibodies:
Rabbit Recombinant Monoclonal MLH1 ab223844 (0.6 μg were used to immobilize the MLH1-MLH3 complex), clone EPR20522, Lot#1014917-2 (Abcam).
Mouse monoclonal Anti-maltose binding protein (MBP) MO913, clone 1612, Lot#017 (MBL) was used for the detection of FAN1 variants (dilution 1:500).
Mouse monoclonal Anti-FLAG F3165, clone M2, Lot#SLCC4005 (Sigma) was used for the detection of FLAG-tagged MLH1 and MSH2 (dilution 1:1000).
In Fig. 4a, for the protein interaction assay we have used the following antibodies:
Mouse Polyclonal Anti-FAN1 ab68572 (Abcam) was used for the detection of FAN1 from soluble extract (dilution 1:500).
Mouse monoclonal Anti-RFC3 (G-10 sc-390293), Lot#E1216 (Santa Cruz Biotechnology) was used for the detection of RFC3 (dilution 1:500).
Rabbit Polyclonal Anti-6x His Tag (PA-983B), Lot#YK385109 (Invitrogen) was used for the detection of His-tagged PCNA (dilution 1:1000)

Validation

Anti-MLH1 antibody's specificity was validated by detecting a signal for MLH1 only in the lanes where recombinant MLH1-MLH3 was added, in Supplementary Figure 8i. According to manufacturer instruction the antibody is suitable and was tested in protein interaction assays and has been Knock-out validated. Anti-MBP specificity was validated in Fig. 6b by detecting both MBP-tagged FAN1 variants where the two recombinants protein were loaded equally as input.
Anti-FLAG antibody's specificity toward MSH2 was validated by detecting a single strong band at the expected molecular weight of 110 KDa in the lanes where recombinant MSH2-MSH3 was added, and only in the presence of MLH1-MLH3, as observed in Figure 6b. The same antibody was used to detect MLH1 and the specificity was validated by detecting a single strong band at the expected molecular weight of 85 kDa only in the lanes where recombinant MLH1-MLH3 is added to the sample. Anti-RFC3 antibody's specificity toward RFC3 was validated by detecting a single strong band at the expected molecular weight of 40 KDa in the lanes where recombinant RFC was added, and only in the presence of MBP-tagged FAN1, as observed in Figure 4a. Anti-6x His Tag antibody's specificity toward PCNA was validated by detecting a single strong band at the expected molecular weight of 30 kDa in the lanes where recombinant PCNA was added, and only in the presence of MBP-tagged FAN1, as observed in Figure 4a.

Eukaryotic cell lines

Policy information about cell lines and Sex and Gender in Research

Cell line source(s)

We used Sf9 cells adapted for suspension growth, available from the cell line collection of the Institute of Molecular Cancer Research, University of Zurich

Authentication

The cell line was not authenticated.

Mycoplasma contamination

The cell line was not tested for mycoplasma contamination.

Commonly misidentified lines
(See ICLAC register)

No misidentified cell lines were used.

Plants

Seed stocks

Report on the source of all seed stocks or other plant material used. If applicable, state the seed stock centre and catalogue number. If plant specimens were collected from the field, describe the collection location, date and sampling procedures.

Novel plant genotypes

Describe the methods by which all novel plant genotypes were produced. This includes those generated by transgenic approaches, gene editing, chemical/radiation-based mutagenesis and hybridization. For transgenic lines, describe the transformation method, the number of independent lines analyzed and the generation upon which experiments were performed. For gene-edited lines, describe the editor used, the endogenous sequence targeted for editing, the targeting guide RNA sequence (if applicable) and how the editor was applied.

Authentication

Describe any authentication procedures for each seed stock used or novel genotype generated. Describe any experiments used to assess the effect of a mutation and, where applicable, how potential secondary effects (e.g. second site T-DNA insertions, mosiacism, off-target gene editing) were examined.
